# Abstract of Meteorological Observations for August, 1854, Made at Philadelphia, Pa.

**Published:** 1854-10

**Authors:** James A. Kirkpatrick


*Abstract of Meteorological Observations for August,* 1854, *made at Philadelphia, Pa. Latitude* 39° 57′ 28″ *N., Longitude* 75° 10′ 40″ *W. from Greenwich.* By PROF. JAMES A. KIRKPATRICK.

| 1854. Aug. | Barometer. Daily Mean | Barometer. Mean Daily Range. | Thermom. Daily Mean | Thermom. Mean Daily Range | Dew Point 2 P M. | Rain. | Prevailing Winds. | General Remarks. |
|---|---|---|---|---|---|---|---|---|
| | Inches. | Inches. | Deg. | Deg. | Deg. | Inch. | Points. | |
| 1 | 29.925 | .083 | 84.0 | 5.7 | 71.0 | 0.018 | SW. | Clear; *rain* previous night. |
| 2 | 29.796 | .129 | 83.5 | 5.7 | 60.0 | 0.058 | SW.N. | M. and ev. clear; aft. cloudy, with *rain.* |
| 3 | 29.883 | .087 | 75.0 | 6.3 | 54.0 | | (Var.) | Clear. |
| 4 | 29.720 | .164 | 79.0 | 4.3 | 60.3 | | W. | M.&A. cl'y; E. cl'r. *Bar. lowest* 29.701 in. |
| 5 | 29.793 | .074 | 78.0 | 2.0 | 53.3 | | W. | Clear. |
| 6 | 29.818 | .025 | 80.0 | 1.2 | 49.7 | 0.210 | NW.W. | M. & A. cl'r; E. cl'dy, with *rain*, th. lt'g. |
| 7 | 29.913 | .095 | 66.0 | 13.2 | 51.3 | 0.040 | W. | Cloudy; aft. showery. |
| 8 | 30.029 | .116 | 65.5 | 6.7 | 49.3 | | N. | Clear. *Therm. lowest* 56°. |
| 9 | 30.050 | .022 | 70.0 | 3.3 | 46.3 | | NE. | Clear. |
| 10 | 30.021 | .030 | 68.0 | 2.0 | 61.3 | | NE. | M. and aft. cloudy; ev. clear. |
| 11 | 29.981 | .053 | 72.0 | 4.5 | 62.7 | | NE.SE. | Cloudy. |
| 12 | 29.918 | .063 | 80.0 | 6.8 | 67.3 | 0.012 | SW. | Cloudy; ev. and night drizzling. |
| 13 | 29.793 | .125 | 83.0 | 2.7 | 57.3 | | SW. | M. cloudy; aft. and evening clear. |
| 14 | 29.921 | .128 | 77.0 | 6.3 | 50.3 | | NW. | Clear. |
| 15 | 29.874 | .109 | 75.5 | 3.7 | 64.0 | 0.005 | (Var.) | M. hazy; aft. cloudy and driz'g; ev. clear. |
| 16 | 29.902 | .073 | 75.0 | 3.0 | 45.7 | | NW.SW | Clear. |
| 17 | 29.916 | .015 | 72.5 | 4.7 | 61.0 | 0.005 | (Var.) | M. drizzling; aft. cloudy; ev. clear. |
| 18 | 29.991 | .074 | 71.0 | 5.3 | 52.0 | | (Var.) | M. and aft. clear; ev. cloudy. |
| 19 | 30.022 | .033 | 75.5 | 2.0 | 60.0 | | NE. | M. and aft. cloudy; ev. clear. |
| 20 | 29.946 | .076 | 76.0 | 3.0 | 54.7 | | SW. | Clear. |
| 21 | 29.982 | .052 | 78.5 | 3.0 | 53.3 | | SE. | M. and aft. cloudy; ev. clear. |
| 22 | 29.902 | .080 | 84.0 | 4.7 | 55.7 | | SW. | M. clear; A. & E. cloudy, th lt'g. *Therm.* |
| 23 | 30.059 | .157 | 73.5 | 10.3 | 51.3 | | NW.E.. | Cloudy. [*hig'st* 95°. |
| 24 | 29.938 | .133 | 73.5 | 2.3 | 63.7 | | SW.S. | Cloudy; very heavy dew at night. |
| 25 | 29.923 | .092 | 82.5 | 6.7 | 59.0 | | (Var.) | Cloudy. |
| 26 | 29.910 | .044 | 81.0 | 5.0 | 62.0 | 0.560 | (Var.) | Cloudy; ev. rain, th. lt'g. and high wind. |
| 27 | 29.908 | .070 | 79.5 | 2.0 | 67.0 | 0.010 | NE. | Cloudy; aft. shower and then drizzling. |
| 28 | 30.124 | .215 | 70.0 | 5.0 | 56.7 | | NE. | Cloudy. |
| 29 | 30.169 | .046 | 67.5 | 3.3 | 51.7 | | NE. | Clear |
| 30 | 30.135 | .038 | 67.0 | 1.0 | 46.7 | | (Var.) | Clear. *Barom. highest* 30.185 in. |
| 31 | 30.074 | .058 | 76.0 | 8.0 | 59.0 | | W. | Cloudy; very heavy dew at night. |
| Means for Aug. 1854 | 29.946 | .083 | 75.5 | 4.6 | 56.7 | 0.918 | N. 81° W. 21—100. | |
| Means for Aug. 1853 | 29.913 | | 74.8 | | 59.3 | 3.080 | | |
| Means for Aug. 1852 | 29.952 | | 72.7 | | 55.3 | 4.400 | | |
| Means for Aug. 3 years | 29.937 | | 74.3 | | 57.1 | 2.799 | W. 17—100. | |

The extreme range of the barometer during the month was 0.484 of an inch, and of the thermometer 39°.

